# Features of interactions responsible for antifungal activity against resistant type cytochrome bc1: A data-driven analysis based on the binding free energy at the atomic level

**DOI:** 10.1371/journal.pone.0207673

**Published:** 2018-11-19

**Authors:** Akihiko Arakawa, Yukako Kasai, Kazuto Yamazaki, Fukumatsu Iwahashi

**Affiliations:** 1 Research Division, Sumitomo Dainippon Pharma Co., Ltd., Konohana-ku, Osaka, Japan; 2 Health & Crop Sciences Research Laboratory, Sumitomo Chemical Co., Ltd., Takarazuka City, Hyogo, Japan; Universidade Nova de Lisboa Instituto de Tecnologia Quimica e Biologica, PORTUGAL

## Abstract

Quinone outside inhibitors (QoIs), which inhibit the mitochondrial respiratory system by binding to the Qo site of Complex III in fungi, are widely used as pesticides with broad spectrum antifungal activity. However, excessive use of QoIs leads to pesticide resistance through mutation of amino acid residues in the Qo site. Recently, metyltetraprole, a novel QoI that is effective against wild-type and resistant mutant fungi, was developed. Interestingly, metyltetraprole has a very similar structure to other QoIs, azoxystrobin and pyraclostrobin, which do not act on resistant mutants. However, it is unknown how slight structural differences in these inhibitors alter their effectiveness towards fungi with amino acid mutations in the Qo site of Complex III. Therefore, we studied the features of interactions of inhibitors effective towards resistant mutants by quantitatively comparing the interaction profiles of three QoIs at the atomic level. First, we reproduced the binding affinity by the thermodynamic integration (TI) method, which treated explicitly environmental molecules and considered the pseudo-binding pathway. As such, a good correlation (R^2^ = 0.74) was observed between the binding free energy calculated using the TI method and experimentally observed pIC_50_ value in 12 inhibitor-target pairs, including wild-type and mutant Complex III in two fungal species, *Zymoseptoria tritici* and *Pyrenophora teres*. Trajectory analysis of this TI calculation revealed that the effectiveness against resistant mutant fungi strongly depended on the interaction of constituent parts of the inhibitor disposed near the active center of the target protein. Specifically, the key in the effectiveness against resistant mutant fungi is that the corresponding component part, tetrazolinone moiety of metyltetraprole, traded off Coulomb and van der Waals interactions in response to subtle changes in the binding pose.

## Introduction

Since the global population has been predicted to grow, a stable supply of grain is important. One of the factors that limit the stable supply of grains is plant disease, which decreases yield and quality. Therefore, various fungicides have been developed and used worldwide to control pathogenic fungi. However, control measures dependent on certain fungicides can lead to increase in resistant fungi and significantly reduce effectiveness of fungicides. To avoid this situation, countermeasures have been applied to use cyclically or mix multiple disinfectants with different mechanisms of action [1]. However, fungicides with a broad action spectrum are intensively used because multiple diseases occur simultaneously in practical situations. Only three classes of fungicides, sterol biosynthesis inhibitors, quinone outside inhibitors (QoI), and succinate dehydrogenase inhibitors exceeded 60% of the disinfectant sales shares in 2016 [2].

**Fig 1:**
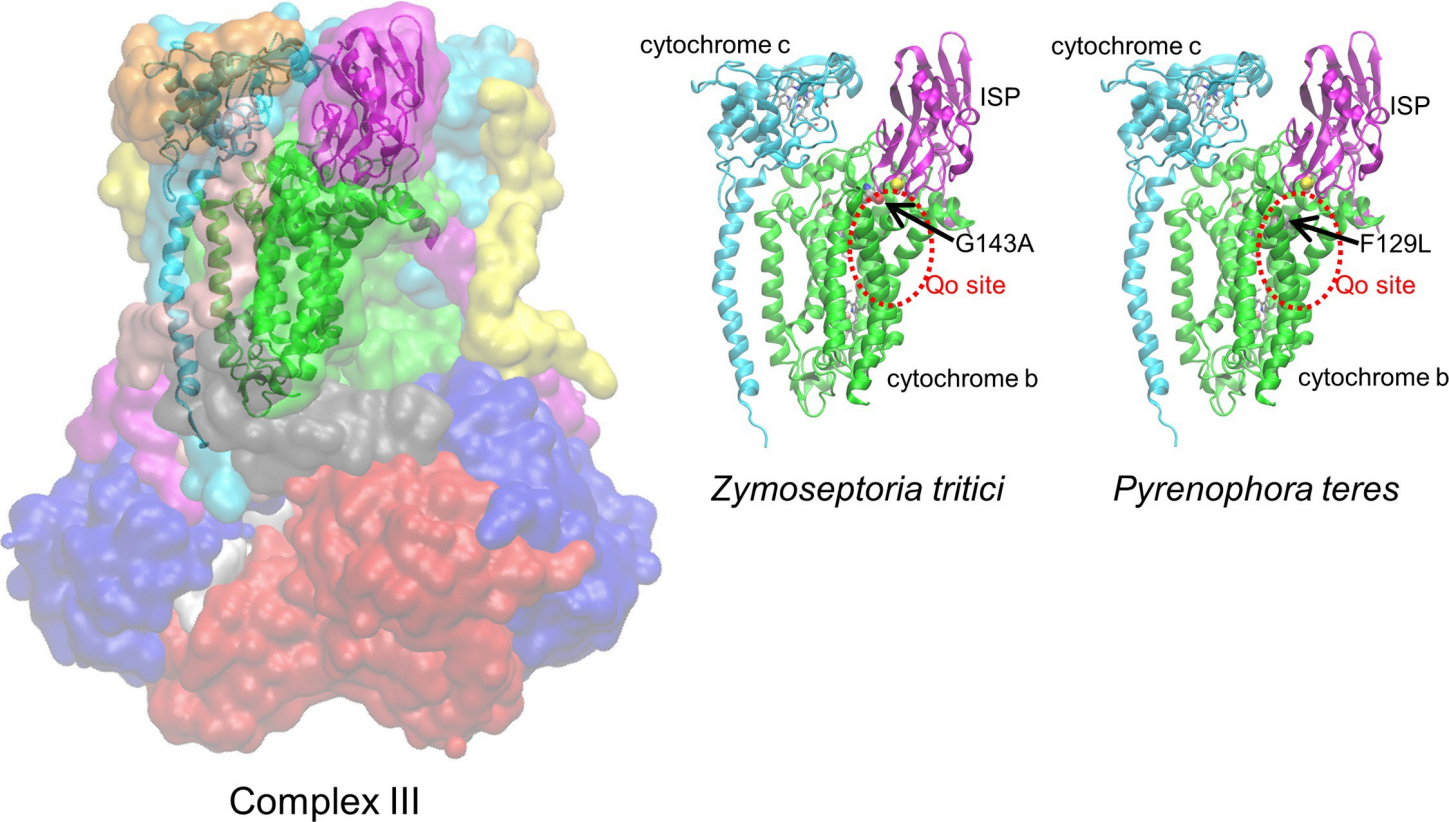
Crystal structure of Complex III and the 3D complex models constructed as the target protein for binding free energy calculation. The crystal structure of bovine Complex III (PDB ID: 2A06) is shown on the left side with separate colors for each subunit. The complex models of *Zymoseptoria tritici* and *Pyrenophora teres* are shown on the center and right side respectively. On these figures, cytochrome b is indicated by green color, cytochrome c1 by cyan color, and ISP by magenta color. Gray-based stick models represent HEM. Pink and yellow sphere models indicate Fe and S atoms, respectively. Red broken lines indicate the Qo sites. The arrowed sphere models indicate the mutant sites.

QoI has been used as a broad-spectrum agent since the 1990s and is currently widely used [3]. Because of this, resistant fungi have been reported in several instances and reduced antifungal activity against some diseases has been confirmed [4]. QoI inhibits the mitochondrial respiratory system by binding to the Qo site of Complex III to generate resistant fungi in which a mutation has occurred in the amino acid residues constituting the Qo site [5]. Interestingly, the mutations in QoI-resistant fungi occur at G143A or F129L of cytochrome b constituting Complex III, regardless of the fungal species (Fig 1) [5].

**Fig 2:**
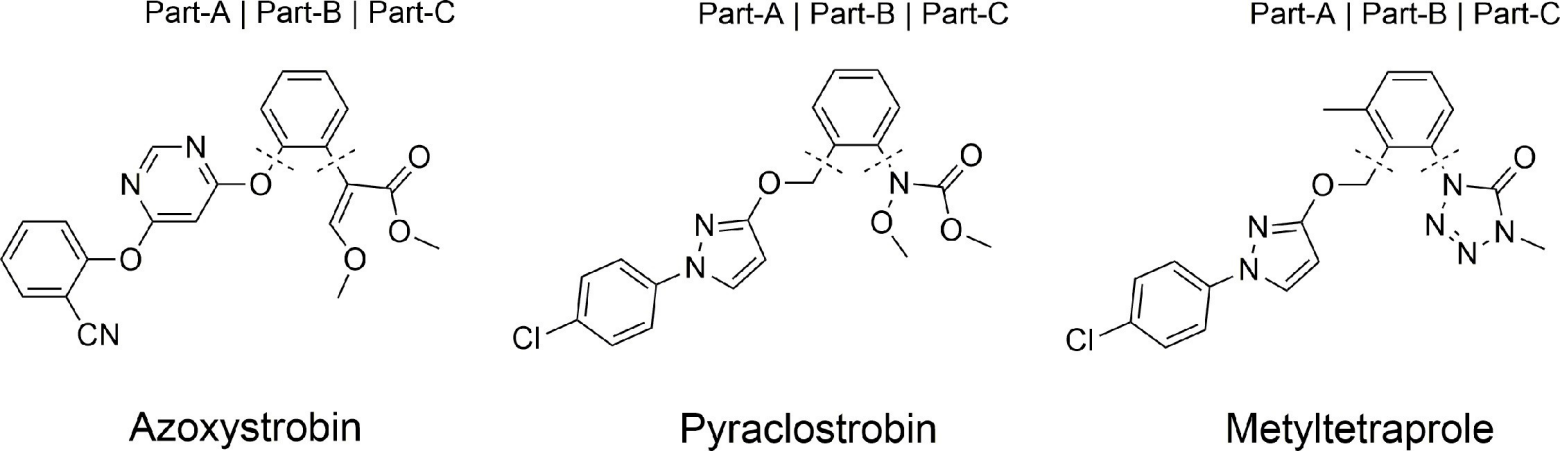
Chemical structure and constituent part of QoIs.

Recently, metyltetraprole, a novel QoI effective against such resistant mutant fungi as well as wild-type fungi, was developed [6]. Interestingly, the chemical structure of metyltetraprole is very similar to those of azoxystrobin and pyraclostrobin [7], which are also QoIs with attenuated action against resistant mutant fungi (Fig 2). How the slight structural differences in these inhibitors avoid the influence of amino acid mutations in the Qo site of Complex III is unknown. If the mechanism of avoidance is clarified, studies exploring novel QoIs such as metyltetraprole effective against resistant fungi can be developed.

A powerful approach for determining the mechanism of avoidance is to compare the interaction profiles of effective and ineffective inhibitors for resistant mutant fungi. This comparison can reveal various features of the interactions inherent in an inhibitor effective for resistant mutant fungi. The interaction profile between the inhibitor and target protein can be obtained by intermolecular affinity determination methods such as surface plasmon resonance and isothermal titration calorimetry. The interaction profile obtained using these methods is a quantitative value of thermodynamics, but it is only the sum of interactions among all atoms. In contrast, X-ray crystallography and nuclear magnetic resonance spectroscopy can reveal interaction profiles at the atomic level, but only provide qualitative information. Thus, it is difficult to experimentally determine an interaction profile that is compatible with quantitative performance and atomic resolution. Molecular simulation can overcome the limits of these experimental methods by generating a detailed interaction profile compatible with quantitative performance and atomic resolution for the binding phenomenon between the inhibitor and target protein.

A previous study reported the interaction of fungicides on the Qo site of Complex III evaluated by molecular simulation [8]. In the previous study, Zhu et al. approximately simulated the binding affinity of ametoctradin and its peripheral derivatives to the Qo site using the molecular mechanics Poisson-Boltzmann surface area (MM-PBSA) method. This suggests that it is possible to obtain an interaction profile for QoI with the target protein. The MM-PBSA method used environmental molecules such as solvents as a continuous dielectric model and calculated the binding free energy as the difference between two states of binding and dissociation [9]. Therefore, this method is effective for evaluating the relative binding affinity of numerous inhibitors for a single target protein at a low calculation cost. On the other hand, the thermodynamic integration (TI) method is known as one of thermodynamic pathway calculation methods, which generally can predict the binding free energy more accurately than MM-PBSA [10]. The thermodynamic integration method realizes more precise calculation because environmental molecules are explicitly treated and pseudo binding pathways are considered from the bound to unbound state [11]. Homeyer et al. suggested that MM-PBSA was useful for an initial ranking in drug discovery and TI was for a drug candidate selection based on the results of comparative experiments on several binding free energy calculation methods [12]. In this study, it is essential to quantitatively reproduce the differences in the affinity for wild-type and mutant Complex III to identify the features of interactions with the inhibitor effective against resistant mutant fungi. Therefore, we reproduced the binding affinity using the thermodynamic integration (TI) method.

Binding free energies for the wild-type and mutant Complex III of three QoIs, azoxystrobin, pyraclostrobin, and metyltetraprole, were calculated using the TI method. To ensure the reliability of our simulation, two strains with different amino acid mutation patterns causing drug resistance were evaluated by TI calculation. We then identified the features of interactions of inhibitors effective for mutant-type resistant fungi by quantitatively comparing their interaction profiles at the atomic level using the simulation results.

## Methods

### Construction of Complex III 3D structure

Complex III is composed of multiple subunits including cytochrome b, cytochrome c1, and Rieske iron sulfur protein (ISP) which are responsible for the electron transfer function (Fig 1). According to a crystal structure of bovine Complex III (PDB ID: 2A06) [13], cytochrome c1 and ISP exist near the Qo site of cytochrome b (Fig 1). Therefore, we constructed a 3D complex model composed of cytochrome b, cytochrome c1, and ISP as a target protein for the binding free energy calculation of QoIs. To save computational cost, cytochrome c1 and ISP in the complex model were not full length, but composed of Ser^1^-Ala^236^ and Ser^56^-Gly^196^, respectively, which made contact with cytochrome b (Fig 1).

**Fig 3:**
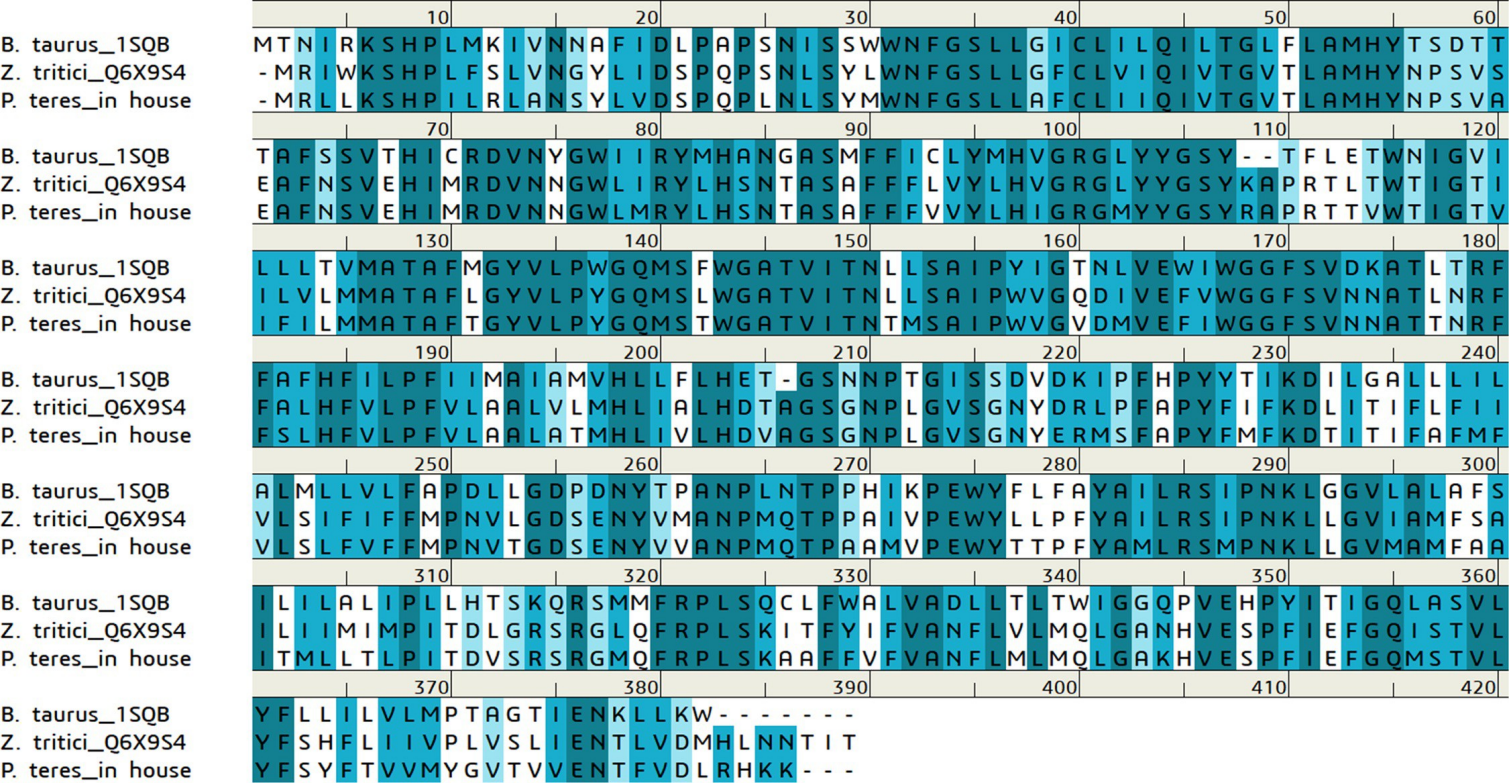
Alignment of amino acid sequences of cytochrome b. Upper is *B*. *taurus* (1SQB) registered in the Protein Data Bank. **Middle is *Z*. *tritici* (Q6X9S4) registered in UniProt. Lower is *P*. *teres* determined through in-house experimentations**.

To build 3D structures of cytochrome b, four types of amino acid sequences were obtained. The amino acid sequence of wild-type *Zymoseptoria tritici* was obtained from UniProt (ID: Q6X9S4). The amino acid sequence of the mutant type was obtained by substituting Ala for Gly^143^ of the wild-type. The wild-type amino acid sequence of *Pyrenophora teres* cytochrome b was determined in-house by full-length cDNA synthesis using the rapid amplification of cDNA end method with fungal genomic DNA as a template, followed by DNA fragment sequencing (Fig 3). The mutant amino acid sequence was obtained by substituting Leu^129^ for Phe^129^. For any type of cytochrome b, only 1–381 residues were subjected to homology modeling (Fig 3). The three-dimensional structure of cytochrome b was constructed by the MODELLER module of Discovery Studio 2017 R2 (BIOVIA) [14] using the crystal structure (PDB ID: 1SQB) of a complex of bovine cytochrome b and azoxystrobin as a template [15]. In this homology modeling, all parameters were set as default values. As for 3D structures of Ser^1^-Ala^236^ of cytochrome c1 and Ser^56^-Gly^196^ of ISP, we used the crystal structure of Complex III, including three heme molecules and an iron-sulfur cluster (PDB ID: 2A06) [13]. The coordinates of Complex III were obtained by root mean square (RMS) fitting of the cytochrome b model structure and cytochrome b/c1/ISP complex crystal structure (PDB ID: 2A06), followed by replacement of the model structure or crystal structure of cytochrome b [13]. Finally, hydrogen atoms were added to each of the constructed complex structures using the Prepare Protein module of Discovery Studio [16].

### Construction of inhibitor-protein complex structure

The binding poses of three inhibitors, metyltetraprole, azoxystrobin, and pyraclostrobin, were analyzed at the Qo site of Complex III. In this analysis, we assumed that the inhibitors generally take a common binding pose for any of the four forms of Complex III. It was also assumed that the three structurally similar inhibitors use mutually similar binding poses. Based on these assumptions, binding poses were analyzed in the bovine Complex III crystal structure bound to azoxystrobin (PDB ID: 1SQB) [15], which was used as a template for homology modeling. In the case of azoxystrobin, the binding pose in this crystal structure was adopted. For a global search of reasonable binding poses of the other two inhibitors, we performed docking studies at the Qo site by the CDOCKER module of Discovery Studio with detailed and efficient searching using the potential energy of the force field as an index [17]. In CDOCKER, all parameters were set as default values, except for “Random Conformations”, which was set to 30 (S1 File). Additionally, the CHARMm [18] force field was adopted for docking analysis. In the case of pyraclostrobin, its lowest potential energy binding pose visually corresponded with the binding pose of azoxystrobin in the crystal structure. Thus, we adopted this binding pose. In contrast, because metyltetraprole could not obtain such a binding pose by docking analysis, its binding pose was obtained by partially editing the crystal structure of azoxystrobin. By combining these binding poses into the four constructed Complex III model structures, 12 pairs of complex structures were constructed.

### Construction and equilibration of simulation system

The complex structure was placed in a box and then filled with TIP3 water models. The box size was set such that the minimum distance between the solute and boundary was 1 nm. Na and Cl ions were added to ensure an electrically neutral state and physiological salt concentration. Energy minimization by the steepest descent method was performed with the position restraint applied to all heavy atoms in the complex until the maximum force decreased to below 100 kJ/mol/nm. The series of simulation system construction processes was archived by GROMACS ver.4.5.5 [19].

The following two-step equilibration was performed on the constructed system. First, molecular dynamics (MD) simulation at the NVT constant (T = 310 K) for 0.1 ns (2 fs per step) was performed with the position restraint applied to all heavy atoms in the complex, after which MD simulation at the NPT constant (P = 101325 Pa and T = 310 K) for 1.2 ns (2 fs per step) was conducted with only the position restraint applied to the Fe2/S2 cluster. In this simulation, CHARMM27 [20] containing CMAP [21] was used as the force field parameter of protein and environmental molecules and CHARMm was applied to the inhibitor and coenzyme. The shake algorithm [22] was applied to all hydrogen atoms except water. Coulomb interactions were calculated by the Particle Mesh Ewald method [23] and a 12-Å cutoff was introduced to calculate van der Waals interactions. A series of equilibration calculations was carried out using MODYLAS ver. 0.9 [24], which provided various physical quantities and MD snapshots. Equilibration was conducted three times with varying initial velocities in the NVT equilibration for each simulation system.

### Calculation of binding free energy by thermodynamic integration method

The coupling parameters in the thermodynamic integration method were set independently for Coulomb force and van der Waals force. The pathway of the coupling parameter (λele, λvdw) was set to 11 states consisting of (1, 1), (0.6, 0.8), (0.4, 0.7), (0.2, 0.6), (0, 0.5), (0, 0.3), (0, 0.2), (0, 0.1), (0, 0.05), (0, 0.02), and (0, 0). For each state, MD simulation at the NPT constant (P = 101,325 Pa and T = 310 K) was performed for 0.2 ns (2 fs per step). Following, 10 snapshots extracted every 0.02 ns were used to integrate the change in internal energy. For the solvation free energy calculation, MD simulation of a system consisting of only the inhibitor in a water environment was performed for 0.04 ns, extracting 10 snapshots every 0.004 ns. We set these parameters of the TI calculation, such as the coupling parameters and simulation time, by referring to the previous study [25] but we did not optimize for Complex III and the QoIs. MD simulation of the coupling pathway was executed by MODYLAS. According to the reported method [26], differentiation of the potential energy involving each inhibitor atom with coupling parameters from the extracted snapshots and integration of the differential values were calculated by a Python script providing the complex and solvation free energies. By subtracting the solvation free energy from the complex free energy, the binding free energy of each inhibitor atom was obtained. Finally, the binding free energy of the whole or a part of an inhibitor was calculated by summing up binding free energies of corresponding atoms. The free energy calculation was repeated three times with varied initial velocities in the NVT equilibration.

## Results & discussion

### Equilibration of simulation system

As described in the Methods section, the simulation system was equilibrated at the NPT constant (P = 101,325 Pa and T = 310 K) prior to the binding free energy calculation. In this equilibration step, confirmation that the simulation system actually reaches an equilibrium state is important in the following two viewpoints. One is to judge whether the constructed simulation system is valid or not, and the other is to judge whether the calculation of binding free energy, which is the next step, is performed correctly. To make these judgements, we confirmed the time transition of total energy and RMSD of an inhibitor. As a result, it was confirmed that the total energy of the simulation system reached a steady state in all simulation systems (S2 File). Further, it was confirmed that the RMSD of the inhibitor from its initial state fluctuated between 1 Å and 2 Å in most simulation systems (S3 File). Even though the RMSD reached 3 Å in some systems, it did not diverge from its initial state and was generally converged (S3 File). Therefore, we concluded that all simulation systems were appropriate for the binding free energy calculation.

As described in the Methods section, the simulation system in this study did not contain all components of Complex III; yet, it contained cytochrome b and fragments of cytochrome c1 and ISP. To investigate how these deletions of constituent proteins affected the equilibrium state of the simulation system, the same equilibrium simulation as above was performed on the complex of azoxystrobin and wild type Z. *tritici* containing all constituent proteins. As a result, the average RMSD of the inhibitor from its initial state was 1.67 Å for 1.2 nsec. Since this value is equivalent to the RMSD in the corresponding simulation system (S3 File), deletion of the constituent proteins was thought to have little influence on the binding pose of the inhibitor. In addition, when comparing the snapshots in the final step of the equilibrium simulation of the target system and the system containing all constituent proteins, no significant difference was observed in both protein and inhibitor (S4 File). These results suggest that the equilibrium obtained from the simulation of the target system in this study is equivalent to the equilibrium state of the system containing all constituent proteins of Complex III.

### Quality of coupling pathway

The TI method calculates binding free energy based on a pseudo-pathway generated by gradually decreasing the coupling parameter. Therefore, it is a necessary condition for a reliable TI calculation that the statistical ensemble of the target system on the pathway is properly sampled. In this study, we adopted a pathway consisting of eleven states whose effectiveness was confirmed in past research [25]. We analyzed the integrated history of binding free energy in the TI calculation for a complex of azoxystrobin and wild type *Z*. *tritici*, one of the target systems, to confirm pathway effectiveness.

In Figure A of S5 File, the values of binding free energy calculated between two states adjacent to each other on the pathway are shown by a line graph. The results of three independent executions with different initial velocities are shown as different lines in this figure. When the number of pathway states and the simulation time of each state are infinitely increased, ideal sampling can be realized. Further the integrated history of the binding free energy is drawn as a smooth curve along the pathway, and all the lines converge to one. The integrated history based on the pathway adopted in this study is not as ideal, and it is recognized that there are non-smooth and variation parts (Figure A in S5 File). However, the line graphs seem to be roughly harmonic curves with tolerable variations (Figure A in S5 File).

On the other side, the feature of this pathway is to simultaneously reduce the Coulomb parameter and the van der Waals parameter in the first half. This is intended to improve computational efficiency, based on empirical knowledge that the state change of the system is small, while decreasing the van der Waals parameter from 1.0 to 0.5. We investigated whether this empirical knowledge was applicable to the target system of this study. For the same complex as above, the TI calculation was performed along a pathway consisting of 15 states, initially decreasing only the Coulomb parameter followed by decreasing the van der Waals parameter. In the integrated history, the binding free energies with van der Waals parameters between 1.0 and 0.5 were almost the same as expected (Figure B in S5 File).

The results of these analyses suggest that it is possible to compute the reliability and efficiency of the target system of this study with the adopted pathway.

### Correlation between experimental binding affinity and calculated binding free energy

Quantitative interaction analysis based on molecular simulation is possible only when the binding affinity of the inhibitors for the target protein is reproduced on a computer. Reproduction of binding affinity was required for both wild-type and mutant type, because we examined the differences in binding affinity of the inhibitor to both types. Moreover, comparative analysis between inhibitors with and without anti-resistant fungal action is indispensable for identifying interaction characteristics that produce anti-resistant fungal action. Therefore, we calculated the binding free energies of three inhibitors, metyltetraprole, azoxystrobin, and pyraclostrobin, for both wild-type and mutant type of Complex III, after which the correlation between the calculated binding free energy and experimental binding affinity was confirmed. For more detailed comparative analysis, this correlation analysis was performed using two different fungal species, *Z*. *tritici* and *P*. *teres*, with different amino acid mutation patterns in Complex III. Unfortunately, binding affinities of the three inhibitors have not been measured experimentally. In contrast, pIC_50_ values of the three inhibitors, which were theoretically correlated with the binding affinities, were reported previously [6]. Thus, we used the pIC_50_ values for the correlation analysis.

**Fig 4:**
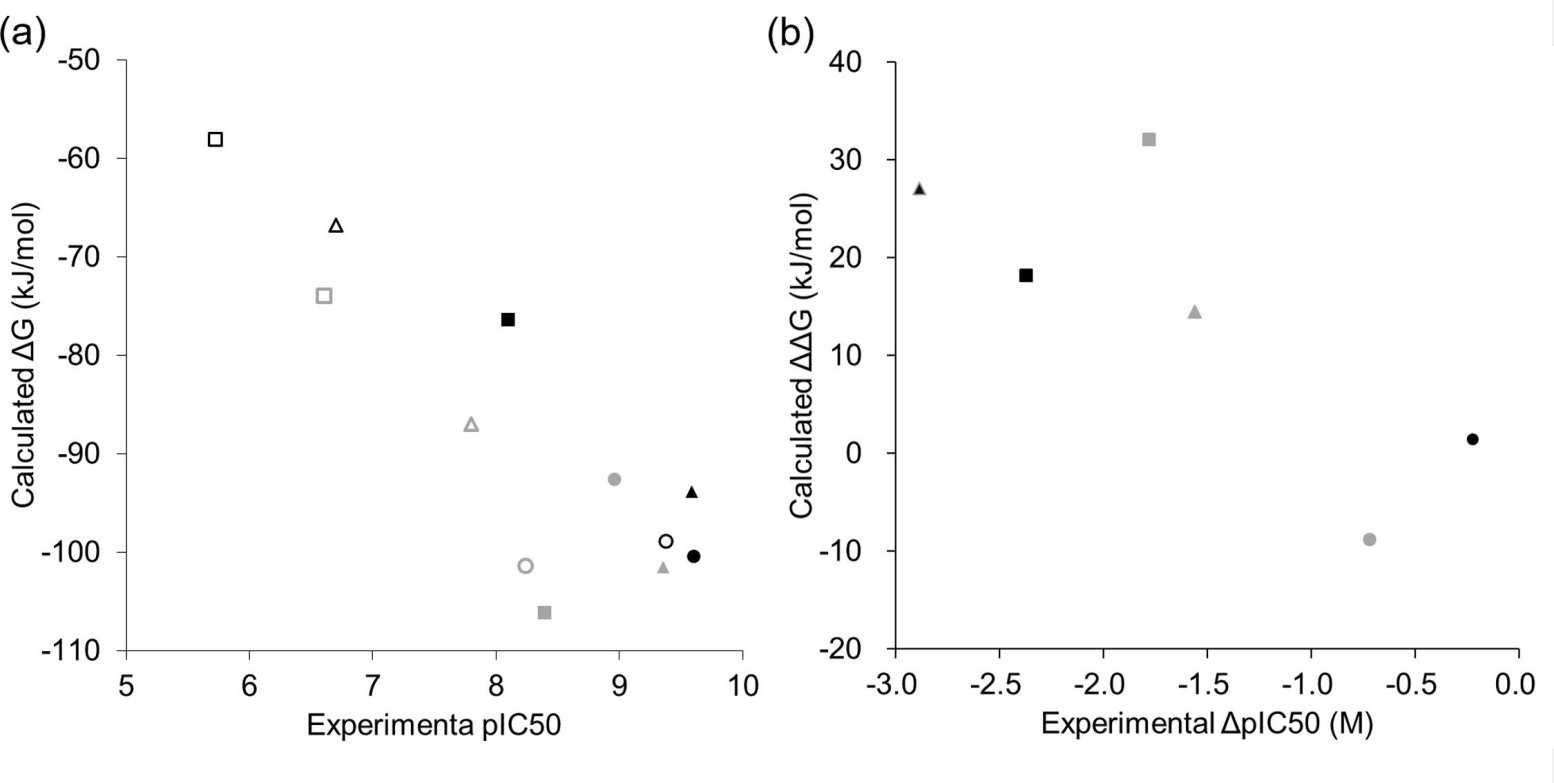
Correlation between experimental binding affinity and calculated binding free energy. (a) Experimental pIC_50_ versus calculated binding free energy ΔG. (b) ΔpIC_50_ and ΔΔG are differences between wild-type and mutant type. *Zymoseptoria tritici* is depicted in black color and *P*. *teres* is in gray color. The symbols indicate the QoIs: azoxystrobin (in square), pyraclostrobin (in triangle), and metyltetraprole (in circle). Wild-type is shown as a closed symbol and mutant type as an open symbol only in (a).

The calculated binding free energies are described in Table 1. Good correlation (R^2^ = 0.74) was observed between the binding free energy calculated from the TI method and experimentally observed pIC_50_ value in all 12 inhibitor-target pairs (Fig 4A). Notably, a wide range of differences in binding affinity was observed for the 12 pairs with only minor differences in constituent atoms. In these correlations, the binding free energy for *P*. *teres* was estimated to be slightly larger than that for *Z*. *tritici*. It is unknown whether this trend is systematic to each target protein or only apparent because of over-estimation of a specific pair. In contrast, examination of the binding affinity difference between the wild-type and mutant type revealed a moderate correlation (R^2^ = 0.62) between the calculated and experimental values (Fig 4B).

**Table 1:** Calculated binding free energy. Average and standard deviation of three calculations.

	*Z*. *tritici*						*P*. *teres*					
	WT			G143A			WT			F129L		
Azoxystrobin	-76.31	±	3.43	-58.05	±	10.91	-106.06	±	8.04	-73.93	±	9.55
Pyraclostrobin	-93.83	±	3.47	-66.74	±	8.61	-101.46	±	5.34	-86.97	±	2.06
Metyltetraprole	-100.36	±	1.07	-98.88	±	1.84	-92.57	±	5.03	-101.34	±	9.28

Unfortunately, a few systems indicated large standard deviation values (Table 1). Moreover, the calculated values did not reproduce the order of six pairs. These were probably because the TI parameters such as coupling parameters and the simulation time, were not optimized for these systems. However, the average value of binding free energy calculated from three simulations showed a good correlation with the experimental value. In particular, the rank correlation and quantitative correlation were reproduced extremely well in terms of fungal type. Thus, we concluded that the simulation results were reliable enough for comparative analysis for each species.

### Binding affinity difference for each constituent of the inhibitor

For comparative analysis based on simulation trajectory, we first analyzed the binding affinity difference between wild-type and mutant target proteins for three constituent parts of the inhibitor as defined in Fig 2.

**Fig 5:**
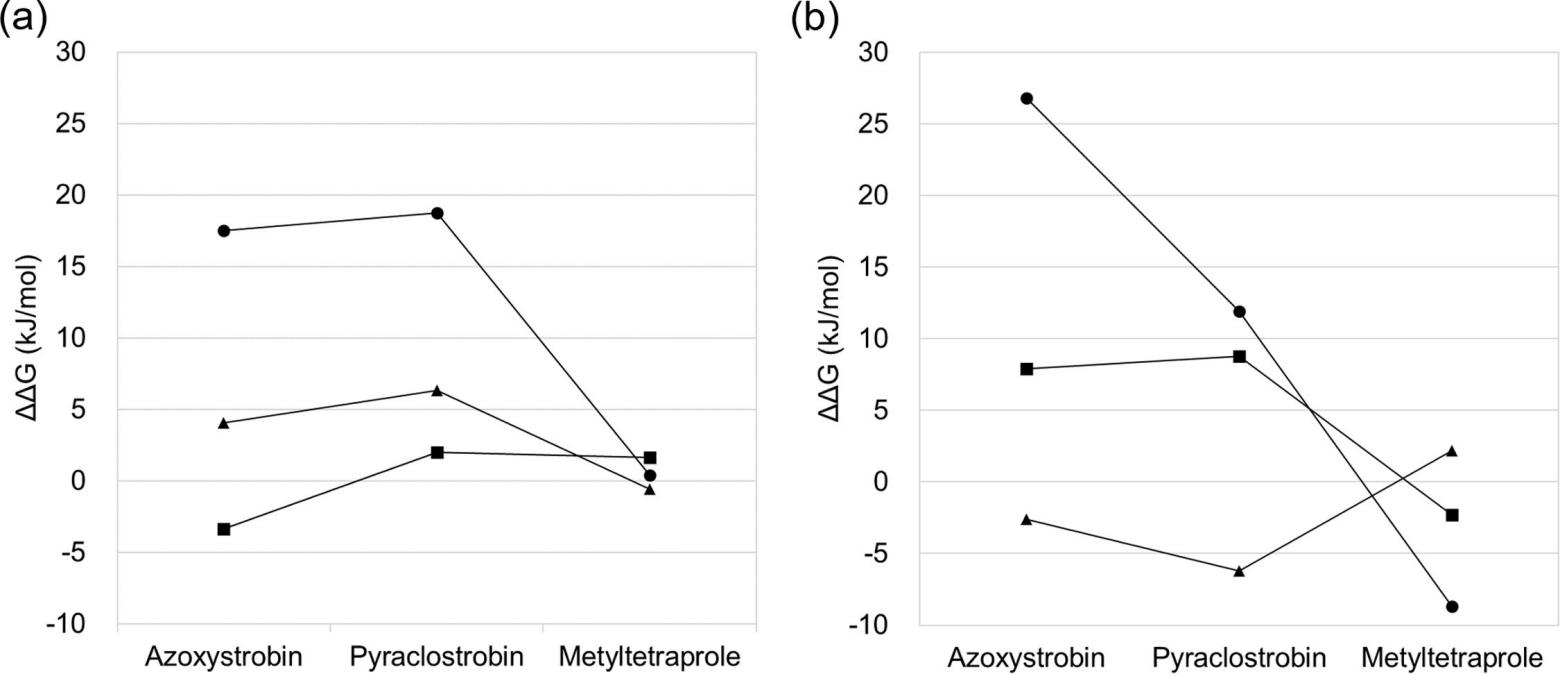
Binding free energy difference between wild-type and mutant type for each constituent part of QoIs. (a) *Zymoseptoria tritici*, (b) *P*. *teres*. The constituent parts are defined in Fig 2 and indicated by different symbols; Part-A by square, Part-B by triangle, and Part-C by circle.

As shown in Fig 5, the binding free energy difference in each constituent part of the three inhibitors is plotted and the same constituent parts are connected by a line. The line graph represents the profile of the binding affinity difference in a constituent part. Overall, azoxystrobin and pyraclostrobin showed a positive difference in binding affinity, while metyltetraprole showed a binding affinity difference near zero. Therefore, a constituent part with a binding affinity difference profile in which azoxystrobin and pyraclostrobin are positive and metyltetraprole is near zero was considered to determine the effectiveness of the inhibitor on the mutant type. From this perspective, Part-B and Part-C for *Z*. *tritici* (Fig 5A) and Part-A and Part-C for *P*. *teres* (Fig 5B) were identified as important structural regions.

Differences in important regions between the two fungal species are derived from differences in the mutation site. That is, Part-B is placed near the mutant G143A of *Z*. *tritici*, whereas Part-A is placed near the mutated F129L of *P*. *teres*. It is reasonable that the constituent parts directly affected by amino acid mutations determine the effectiveness of the mutant type. Interestingly, Part-C, which is distant from the mutated amino acid residues, dominates the effectiveness of the inhibitor for the mutant type rather than the constituent parts near them (Fig 5). Therefore, subsequent comparative analysis was conducted to evaluate the interaction of Part-C.

### Contribution of each constituent part of inhibitor to binding affinity

**Fig 6:**
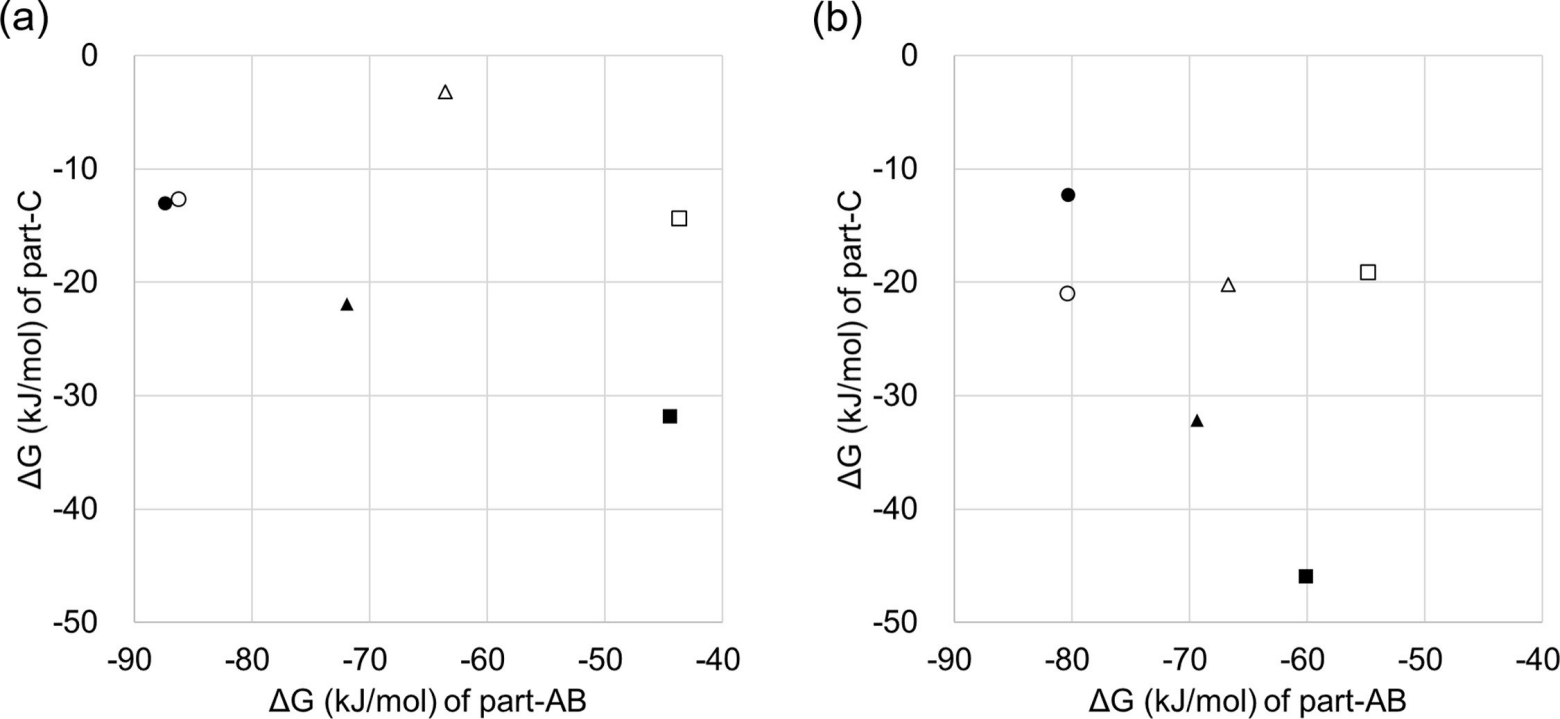
Contribution ratio of Part-AB and Part-C to the ΔG value. (a) *Zymoseptoria tritici*, (b) *P*. *teres*. Three QoIs are shown by different symbols: azoxystrobin, pyraclostrobin, and metyltetraprole are indicated by a square, triangle, and circle, respectively. Closed plot indicates ΔG for wild-type, while an opened plot is shown for mutant type.

Based on the findings described in the previous section, the binding affinities of inhibitors for Complex III were divided into Part-C and other parts (Part-A & B), and then plotted as the binding free energy of Part-A & B versus the binding free energy of Part-C (Fig 6).

First, the contribution of Part-C to the binding affinity for wild-type *Z*. *tritici* generally decreased in the order of azoxystrobin, pyraclostrobin, and metyltetraprole (Fig 6A). This same trend was observed for *P*. *teres* (Fig 6B). In contrast, the contribution of Part-C to the binding affinity for the mutant of both strains was small for all inhibitors. The contribution of Part-A & B was nearly the same in both the wild-type and mutant type. Taken together, these results indicate that as the binding affinity of Part-C for wild-type increases, that for the mutant decreases. Theoretically, strong binding affinity indicates high complementarity with the target, suggesting high position specificity of the interaction. High position specificity of the interaction is thought to be affected by shifts in the binding pose. Therefore, as the binding affinity of Part-C for wild-type increased, that for the mutant decreased.

However, this rationale is currently theoretical; in reality, the correlations between the binding affinity for wild-type of Part-C and difference in binding affinity between the wild-type and mutant are complex. Particularly, in pyraclostrobin for *Z*. *tritici*, although the binding affinity of Part-C for the wild-type is close to that of metyltetraprole, the binding affinity difference between the wild-type and mutant type is equivalent to that of azoxystrobin. Additionally, the binding affinity of Part-C of metyltetraprole for the mutant type of *P*. *teres* is stronger than that for the wild-type. Therefore, additional studies are needed to examine the Part-C interaction.

### Binding affinity component of Part-C

**Fig 7:**
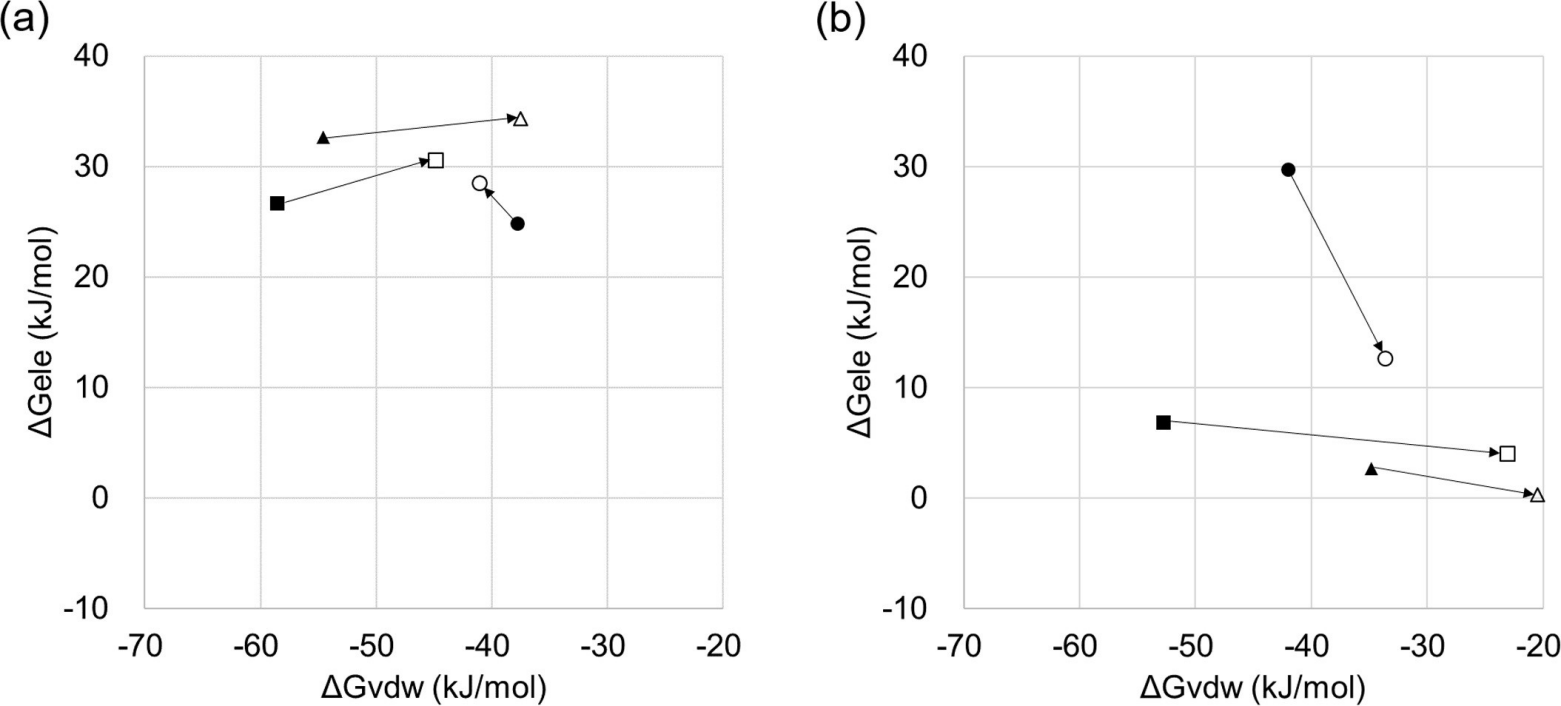
Contribution ratio of van del Waals and Coulomb components for ΔG value of Part-C. (a) *Zymoseptoria tritici*, (b) *P*. *teres*. Three QoIs are shown by different symbols: azoxystrobin by squares, pyraclostrobin by triangles, and metyltetraprole by circles. Closed plot indicates ΔG for wild-type, while open plot indicates mutant type. An arrow is shown from the wild-type to the mutant type of the same inhibitor.

To evaluate the features of the interaction determining the effectiveness of the mutant type, the binding affinity of Part-C was decomposed into van der Waals and Coulomb components and then compared among inhibitors. As shown in Fig 7, three inhibitors were plotted as van der Waals components on the horizontal axis and as Coulomb components on the vertical axis. Importantly, this plot reveals the relative positional relationship between the wild-type and mutant type, as indicated by arrows. In azoxystrobin and pyraclostrobin for *Z*. *tritici*, the difference in the Coulomb component between wild-type and mutant type was small, while the difference in van der Waals component was remarkably large (Fig 7A). This was also observed for *P*. *teres* (Fig 7B). In contrast, for metyltetraprole, the relative positional relationship largely differs from the other inhibitors in both fungal species. Specifically, van der Waals and Coulomb components are arranged diagonally and there is a trade-off relationship. These differences in relative positional relationships appear to represent feature of interactions determining the effectiveness of the mutant type. Thus, an inhibitor that reduces the binding affinity for the mutant type preferentially maintained the Coulomb component in the altered binding state, resulting in attenuation of the van der Waals component. In contrast, an inhibitor that maintains the binding affinity for the mutant type may compensate for the decrease in one component by increasing the other component depending on the environment.

### Insights about features of interactions based on complex structure

Analysis based on the binding free energy revealed that the effectiveness against the mutant type was mainly caused by differences in the interactions of Part-C. Additionally, the interaction of Part-C in the inhibitors effective for the mutant type was relatively weak, with Coulomb and van der Waals interactions in a trade-off relationship as the binding state changed. By comparing these findings with the complex structure in the trajectory of the simulation, we had deeper insights into the features of the interaction effective for the mutant type.

**Fig 8:**
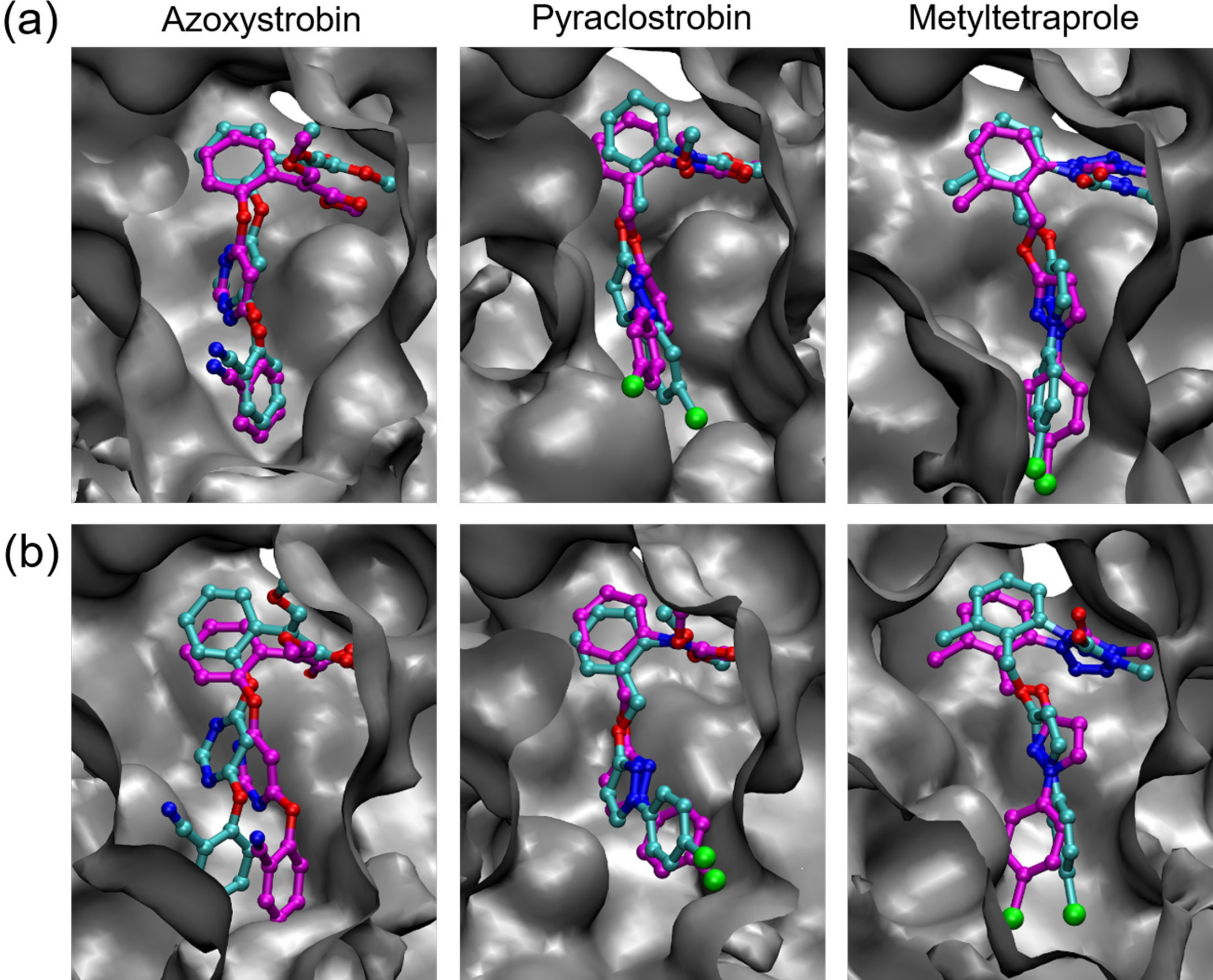
Binding pose of inhibitor in the initial state of TI calculation. Both binding poses for wild-type and mutant type are shown aligned on the active site of wild-type, which is shown in the gray-colored surface model. Cyan colored stick model shows binding pose for wild-type, while magenta-colored model shows those for mutant type. (a) *Zymoseptoria tritici*, (b) *P*. *teres*.

First, comparing the binding poses of inhibitors to wild-type and mutant type, we detected some differences in the inhibitors (Fig 8). However, the differences did not drastically alter the binding position and orientation of the entire molecule but remained at the level of inducible adaptation to amino acid mutations. Interestingly, the component part with the smallest difference in binding pose was Part-C. This represents a reasonable strategy by fungi to invalidate inhibitors, as it is more efficient to break interactions with strong complementarity by slightly changing the binding pose than to drastically alter the binding pose of the inhibitor. Metyltetraprole is effective against the mutant type because its binding affinity to the target does not rely on the corresponding interaction.

**Fig 9:**
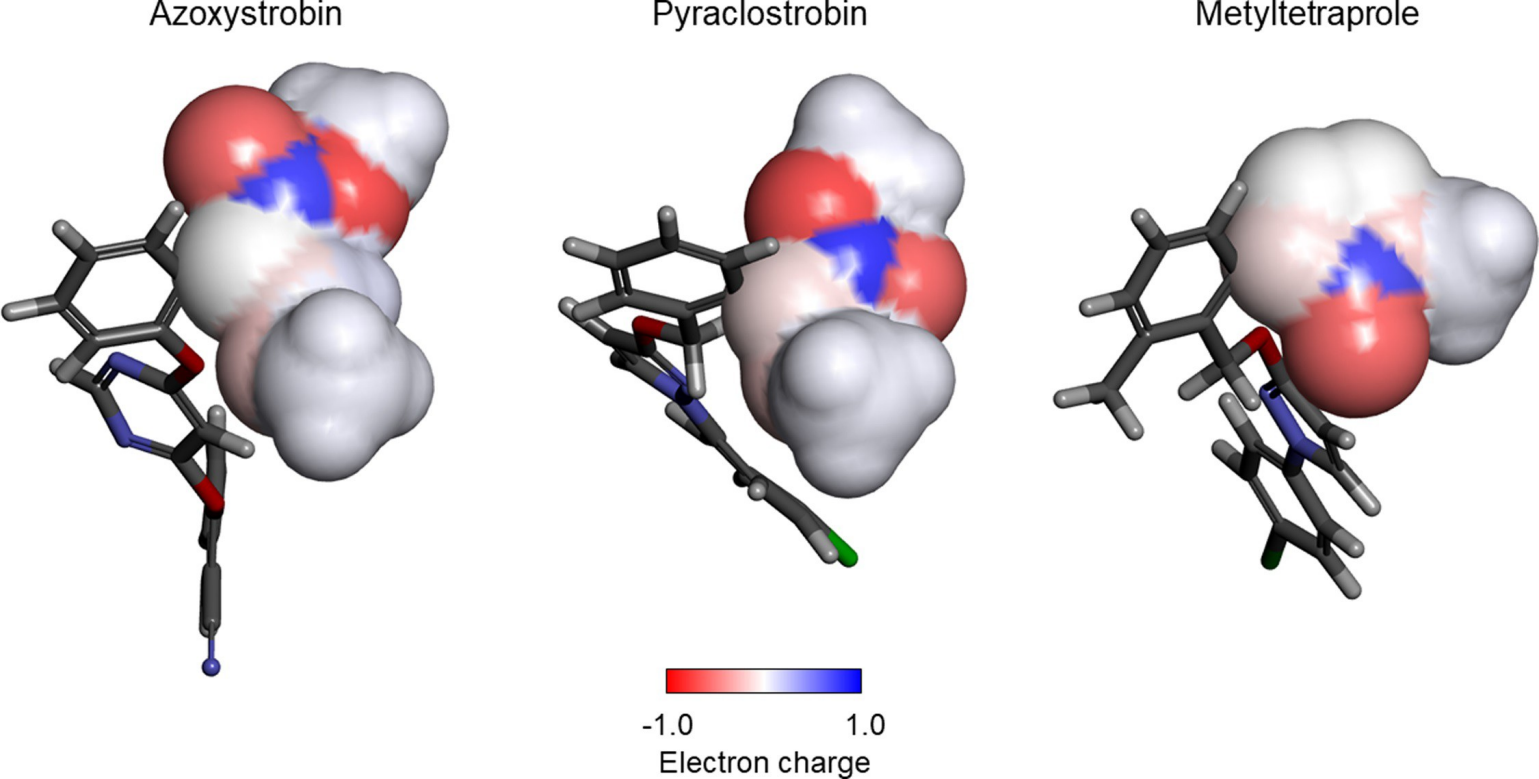
Partial charges and molecular shape of Part-C in QoIs. The molecular shape is shown by a surface model and partial charges by a color gradient on its surface. Stick model shows Part-A and Part-B in the active conformer of QoIs.

Next, we examined how a small change in the binding pose can cause large attenuation of the interaction. Three polar residues of Tyr^132^, Glu^273^, Tyr^275^ are present in the binding site of Part-C. The polar functional groups in these amino acids form a hydrogen bond network; no hydrogen bond formation with the inhibitor was observed in the simulation trajectories. However, Part-C was clearly in a relatively polarized environment. On the other hand, each inhibitor had a relatively strong partial charge in Part-C (Fig 9). Compared to metyltetraprole, azoxystrobin and pyraclostrobin have a wider range of partial charges. Thus, Part-C of azoxystrobin and pyraclostrobin have highly specific positions and orientations in their Coulomb interactions. Fig 9 shows the shape of the molecule with a partial charge. Part-C of azoxystrobin and pyraclostrobin is elliptical, whereas that of metyltetraprole is spherical and compact. The former has higher position and orientation specificity in van der Waals interactions.

In summary, Part C of azoxystrobin and pyraclostrobin must have high specificity in both Coulomb interactions and van der Waals interactions to bind the target. A high specificity in both of these interactions attenuates the affinity because of slight differences in the binding pose. In contrast, Part-C of metyltetraprole with a narrow range of partial charges and spherical and compact shape exhibits some difficultly in forming specific interactions. Therefore, it is considered that trade-offs of both interactions according to the environment are established.

## Conclusions

In this study, it was revealed that Part-C of metyltetraprole having a narrow range of partial charges and a spherical and compact shape produces effectiveness against resistant mutant fungi. And it was also revealed that metyltetraprole is a unique QoI distinct from existing inhibitors due to its interaction with the target. The structural features of Part-C in metyltetraprole may be useful indicators for designing novel QoIs. Furthermore, the molecular simulation protocol used in this study can be used to evaluate the binding affinities for both wild-type and mutant Complex III.

Trajectory analysis of molecular simulation generally focuses on geometry, such as the binding pose and relative arrangements between functional groups. Such geometry-based trajectory analysis is effective if remarkable differences exist between the comparison objects. However, there are two limitations. First, geometry-based analysis can only generate indirect knowledge regarding the binding affinity, as it covers the binding state rather than the binding process. Second, differences in binding affinity created by the accumulation of small interaction differences may lead to false findings. In contrast, the trajectory analysis performed in this study differed from geometry-based analysis. First, we analyzed the direct relationship between the binding affinity and interaction by consistently targeting the binding free energy. Additionally, without putting in hypotheses, we gained insight through a data-driven approach. In other words, data-driven analysis based on binding free energy avoids the limitations of geometry-based analysis. This method is extremely effective for comparative analysis between pairs in which both the target and inhibitor have similar structures.

## Supporting information

S1 File CDOCKER parameters. (XLSX)

S2 File Total energy at the NPT constant (P = 101325 Pa and T = 310 K) equilibration for 1.2 ns. (a) Zymoseptoria tritici, (b) Pyrenophora teres. Three lines of different colors indicate the results of three analyses conducted by changing the initial rate.
(TIF)

S3 File Root mean square distance (RMSD) of each inhibitor in equilibration, which is the MD simulation at the NPT constant (P = 101325 Pa and T = 310 K) for 1.2 ns without constraints. (a) Zymoseptoria tritici, (b) Pyrenophora teres. Three lines of different colors indicate the results of three analyses conducted by changing the initial rate. RMSD was calculated using the distance of all heavy atoms from the average structure of all snapshots.
(TIF)

S4 File Snapshots in the final step of the equilibrium simulation for the complex of azoxystrobin and wild-type Z. tritici. The overall structures are shown on the left side, and the structures near the Qo site are on the right side. In both figures, the simulation system in this study is shown as green color, and the system containing all constitute proteins of Complex III is magenta color. Azoxystrobin is shown as a ball & stick model.
(TIF)

S5 File The integrated history of binding free energy for a complex of azoxystrobin and wild-type S. tritici. The binding free energies between two states adjacent to each other on the pathway are shown as line graphs. Three different lines indicate the results of three analyses conducted by changing the initial rate. (a) coupling pathway consisting of 11 states, (b) coupling pathway consisting of 15 states.
(TIF)
