# Supplementary figures and images for: Features of interactions responsible for antifungal activity against resistant type cytochrome bc1: A data-driven analysis based on the binding free energy at the atomic level
Source: PLoS One. 2018 Nov 19;13(11):e0207673. doi: 10.1371/journal.pone.0207673 (PMC6242680; doi:10.1371/journal.pone.0207673)

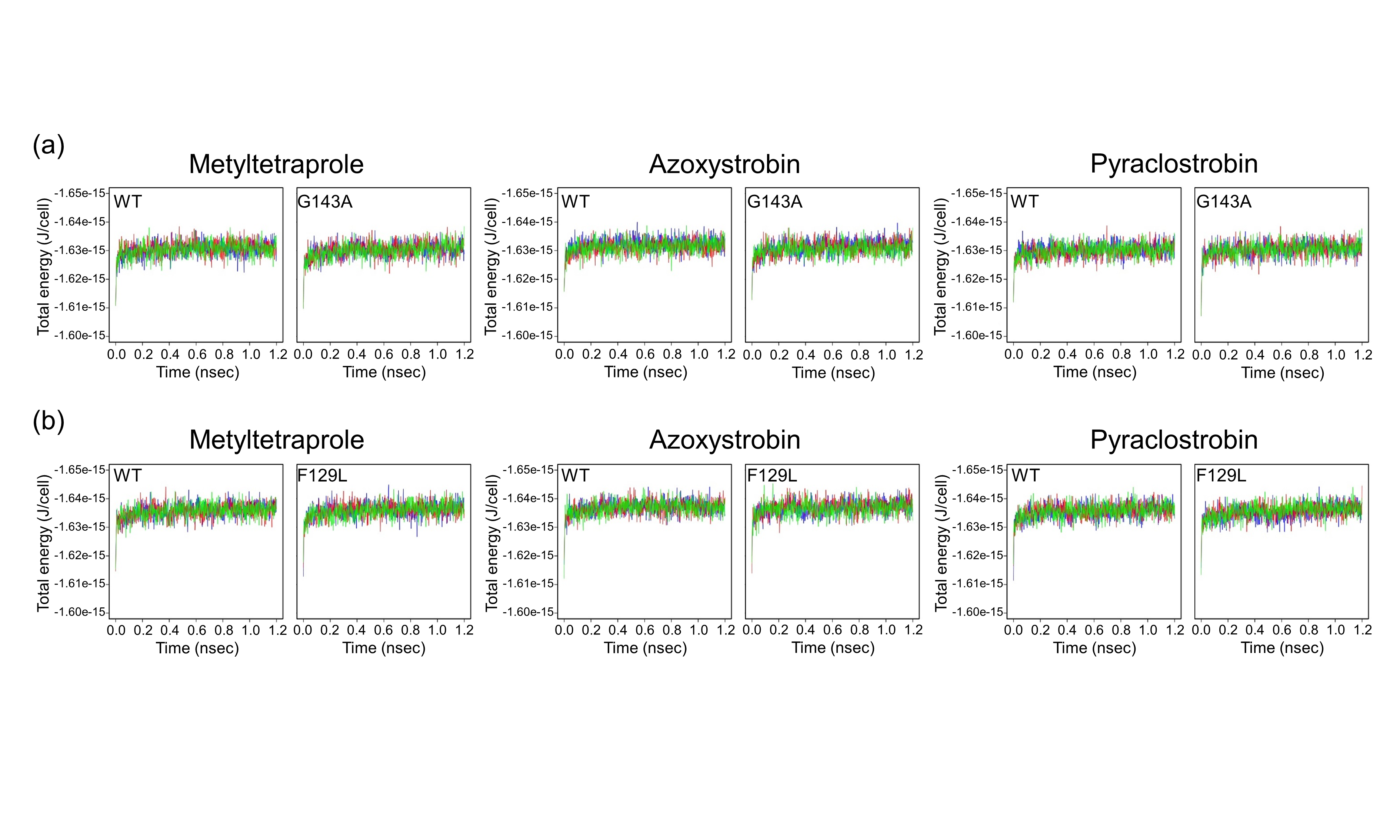

Supplement: S2 File — (a) Zymoseptoria tritici, (b) Pyrenophora teres. Three lines of different colors indicate the results of three analyses conducted by changing the initial rate. (TIF) [file pone.0207673.s002.tif]

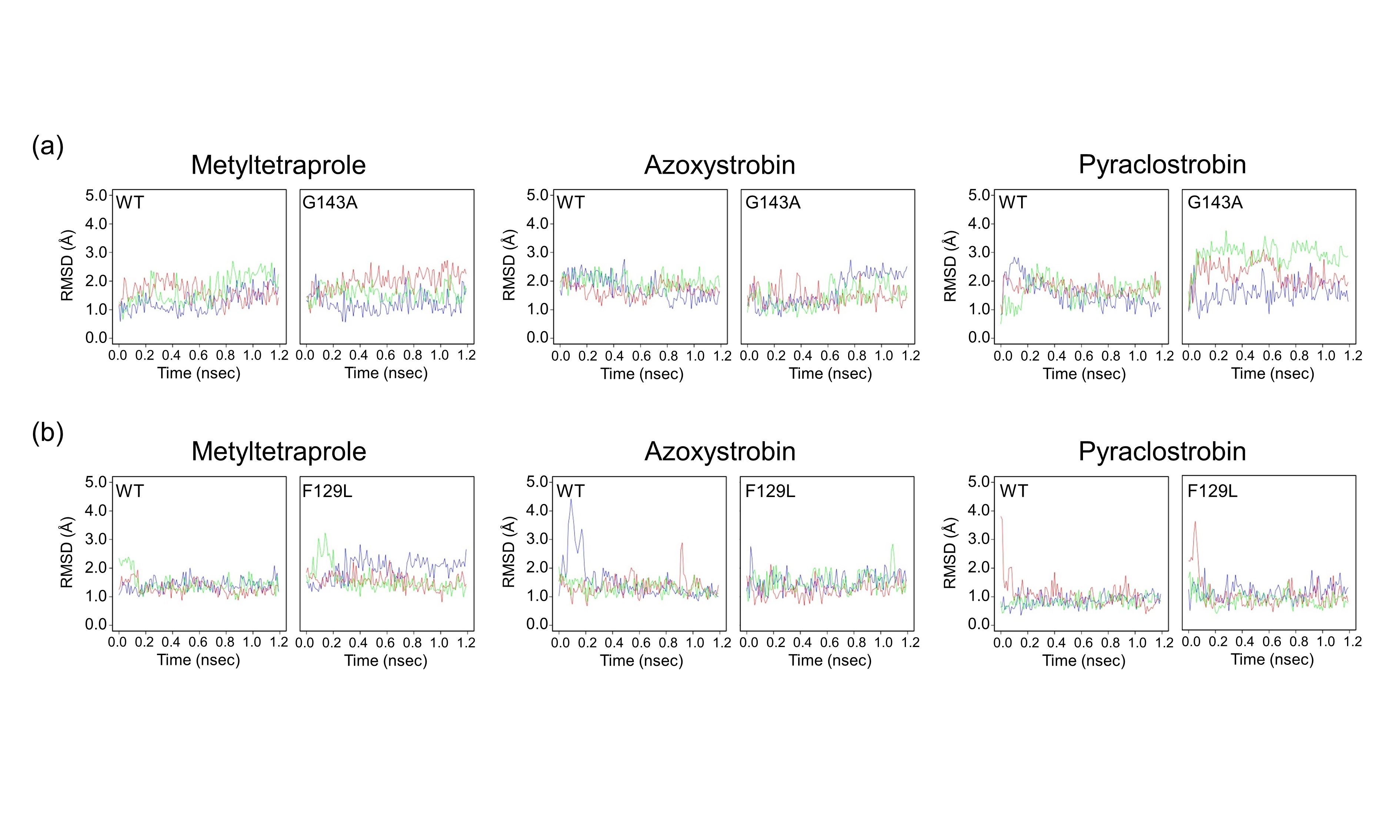

Supplement: S3 File — (a) Zymoseptoria tritici, (b) Pyrenophora teres. Three lines of different colors indicate the results of three analyses conducted by changing the initial rate. RMSD was calculated using the distance of all heavy atoms from the average structure of all snapshots. (TIF) [file pone.0207673.s003.tif]

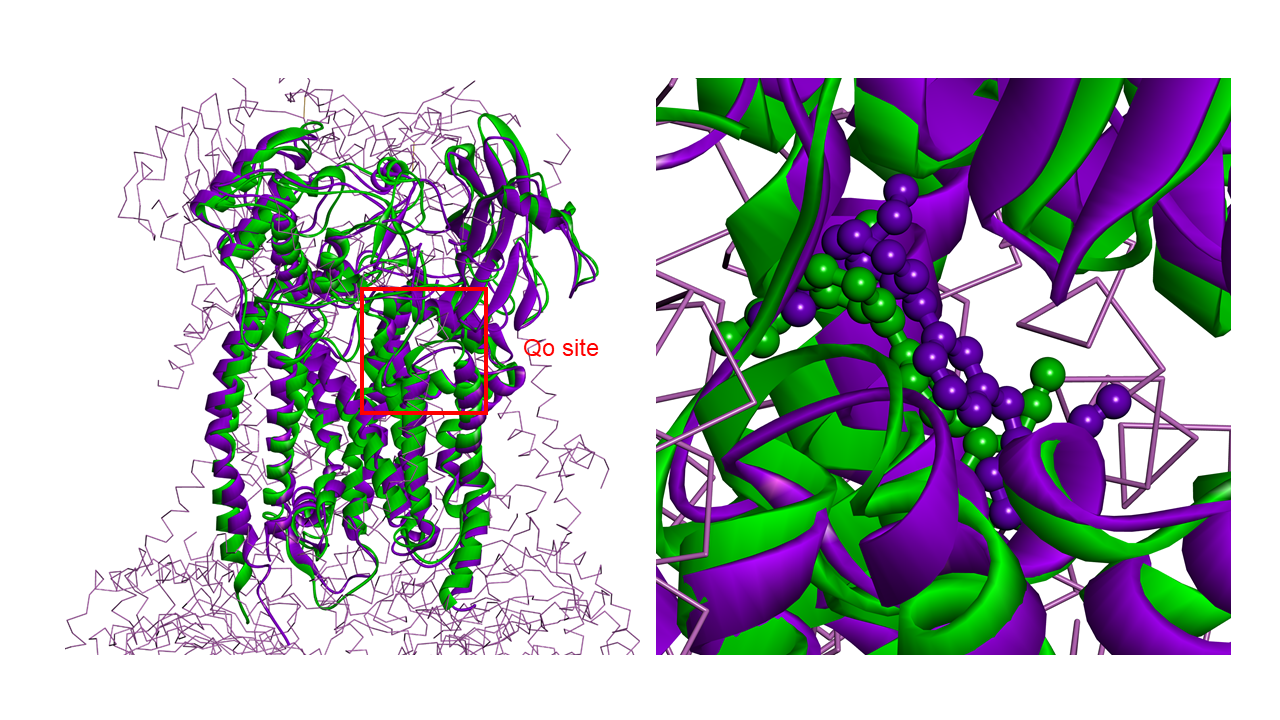

Supplement: S4 File — The overall structures are shown on the left side, and the structures near the Qo site are on the right side. In both figures, the simulation system in this study is shown as green color, and the system containing all constitute proteins of Complex III is magenta color. Azoxystrobin is shown as a ball & stick model. (TIF) [file pone.0207673.s004.tif]

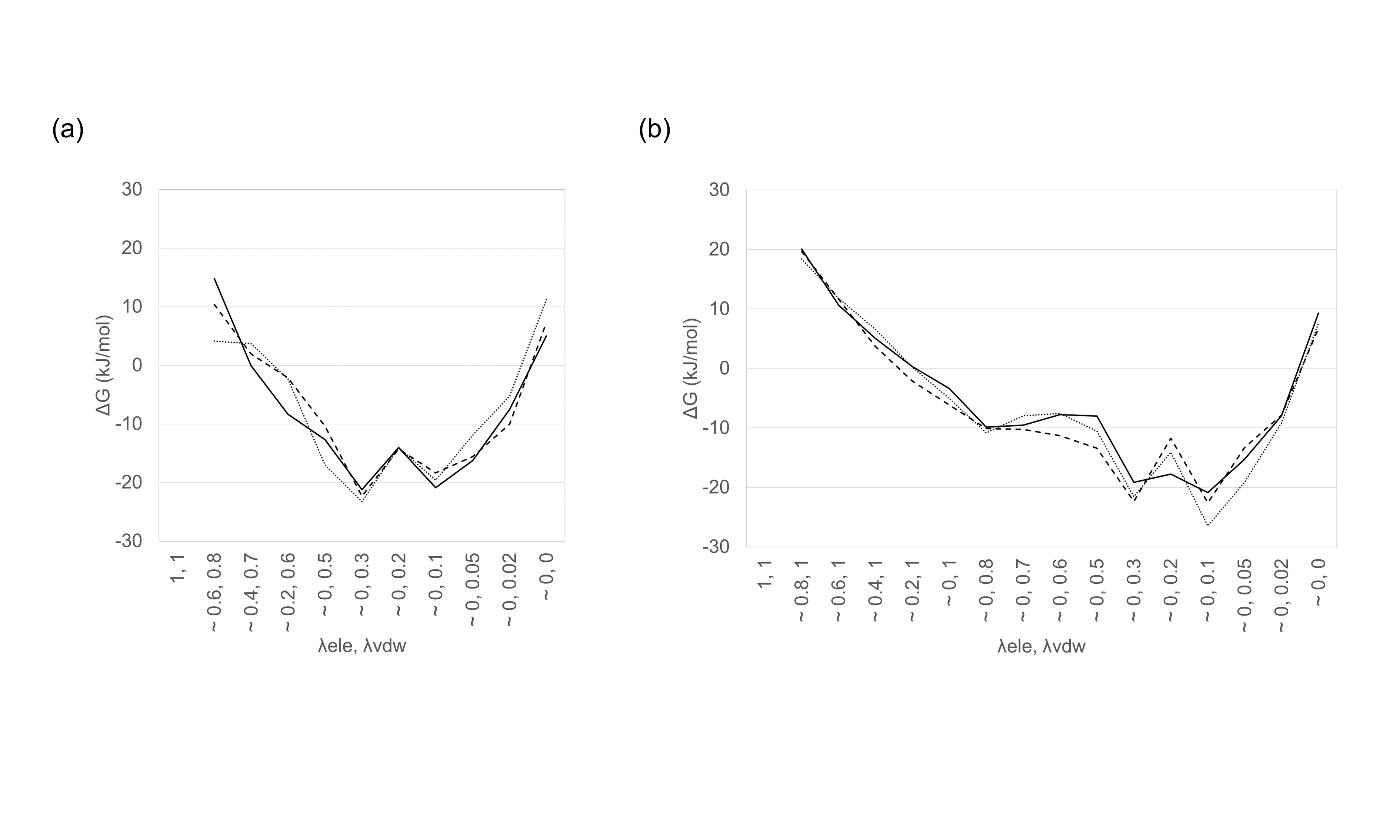

Supplement: S5 File — The binding free energies between two states adjacent to each other on the pathway are shown as line graphs. Three different lines indicate the results of three analyses conducted by changing the initial rate. (a) coupling pathway consisting of 11 states, (b) coupling pathway consisting of 15 states. (TIF) [file pone.0207673.s005.tif]
